# Supplementary material for: A Deep Learning Approach for Improving Two-Photon Vascular Imaging Speeds
Source: Bioengineering (Basel). 2024 Jan 24;11(2):111. doi: 10.3390/bioengineering11020111 (PMC10886311; doi:10.3390/bioengineering11020111)
Supplement: Supplementary file 1 [file bioengineering-11-00111-s001.zip › bioengineering-2733888-supplementary.pdf]

bioengineering

MDPI

Supplemental Figures

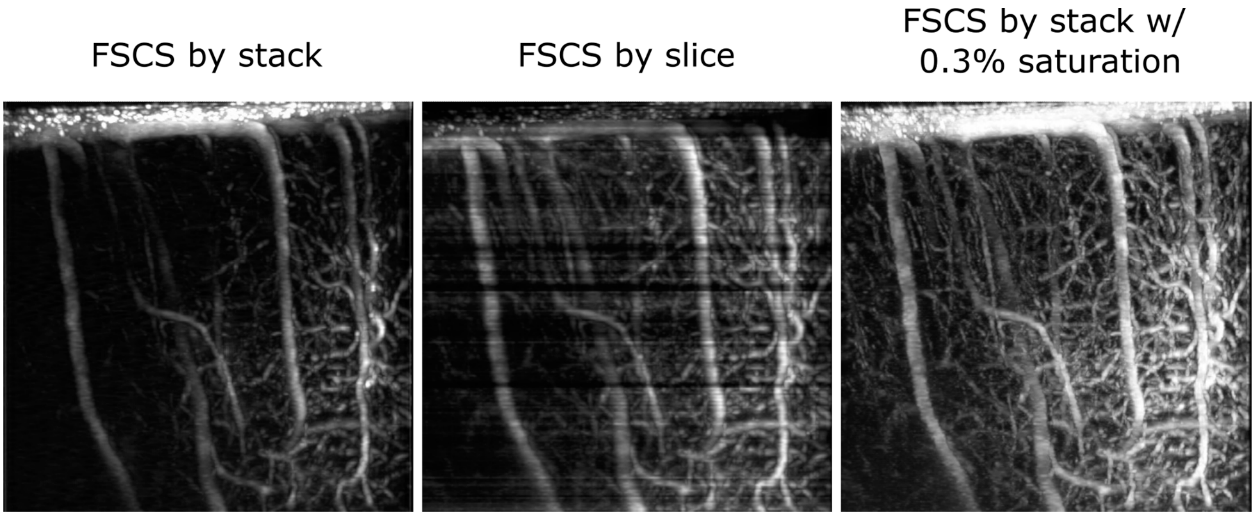

Figure S1. Test image results for models trained & tested with different normalization methods. Sagittal projections of model output image stacks that underwent full scale contrast stretch (FSCS) with respect to the entire stack, per slice, or entire stack with 0.3% saturation prior to input to each respectively trained model as test images. FSCS was done with Fiji ImageJ normalization function[1].

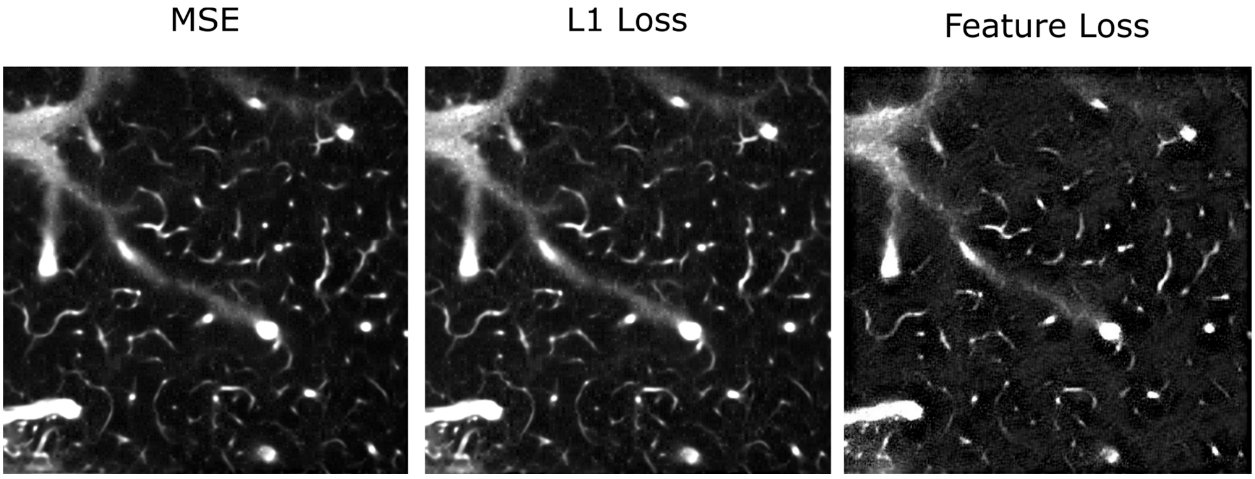

Figure S2. Test image results for models trained with different loss functions (single image slice).

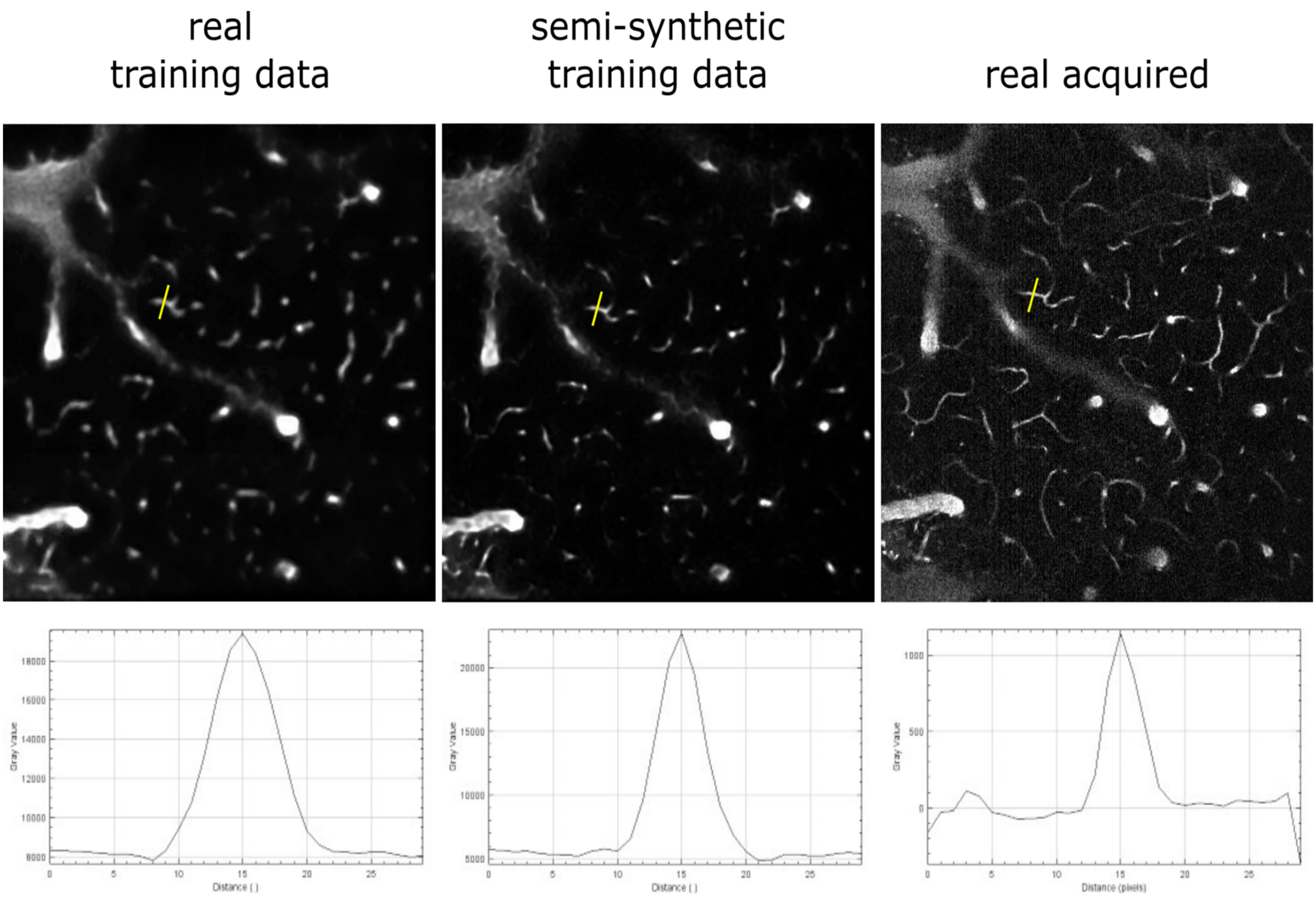

Figure S3. Vessel diameter comparison for test image outputs from models trained with real training data vs. semi-synthetic training data, vs. a real-acquired image. Approximate vessel diameters are as follows in units of pixels: real training: 13, semi-synthetic training: 10, real acquired 7.

References

1. Schindelin, J.; Arganda-Carreras, I.; Frise, E.; Kaynig, V.; Longair, M.; Pietzsch, T.; Preibisch, S.; Rueden, C.; Saalfeld, S.; Schmid, B.; et al. Fiji: An open-source platform for biological-image analysis. *Nat. Methods* **2012**, *9*, 676–682.
